# CA125: a novel cardiac biomarker for infants with congenital diaphragmatic hernia

**DOI:** 10.1038/s41390-022-02130-8

**Published:** 2022-06-15

**Authors:** Lukas Schroeder, Flaminia Pugnaloni, Ramona Dolscheid-Pommerich, Annegret Geipel, Christoph Berg, Stefan Holdenrieder, Andreas Mueller, Florian Kipfmueller

**Affiliations:** 1 Department of Neonatology and Pediatric Intensive Care Medicine, University Children’s Hospital Bonn, Bonn, Germany; 2 Department of Pediatrics, Obstetrics and Gynecology, “Sapienza” University of Rome, Rome, Italy; 3 Institute of Clinical Chemistry and Clinical Pharmacology, University Hospital Bonn, Bonn, Germany; 4 Department of Obstetrics and Prenatal Medicine, University Hospital Bonn, Bonn, Germany; 5 Department of Obstetrics and Prenatal Medicine, University Hospital Cologne, Cologne, Germany; 6 Institute for Laboratory Medicine, German Heart Centre, Technical University Munich, Munich, Germany

## Abstract

**Background:**

The carbohydrate antigen 125 (CA125) was proven as a robust biomarker for risk stratification in adults with heart failure. This is the first study analyzing CA125 in a cohort of infants with congenital diaphragmatic hernia (CDH).

**Methods:**

Sixty-eight infants with CDH, treated at the University Children’s Hospital Bonn (Germany), between January 2018 and February 2021, were prospectively enrolled for analysis. CA125 values were measured at the following timepoints: 6,12, 24, 48 h, and during ECMO daily from day 1 to day 7.

**Results:**

In infants not surviving to discharge, CA125 values were significantly higher at day 1 (6, 12, and 24 h). Infants with subsequent need for ECMO presented significantly higher CA125 values at 12 h of life. During ECMO, CA125 values measured at day 1 were significantly higher in infants not surviving to discharge. In the ROC analysis, a CA125 value of ≥10 U/ml was calculated as optimal cut-off for the prediction of ECMO and in-hospital mortality. CA125 values correlated significantly with the severity of PH and ventricular dysfunction.

**Conclusions:**

CA125 values correlate significantly with echocardiographic markers of PH and ventricular dysfunction and correlate significantly with parameters of disease severity (need for ECMO, mortality).

**Impact:**

- CA125 was proven as robust cardiac biomarker in adult cohorts.
- Information about the utility as a biomarker in neonatal cohorts is lacking.
- This is the first study analyzing CA125 as a cardiac biomarker in a cohort of infants with congenital diaphragmatic hernia (CDH).
- CA125 correlates significantly with markers of echocardiographic assessment (PH and ventricular dysfunction) in infants with CDH and helps to identify infants at high risk for ECMO and in-hospital mortality.
- The results underline the need for the inclusion of cardiac biomarkers in the clinical routine in neonates at risk for cardiopulmonary failure.

## Introduction

Despite decades of investigation and novel therapeutic approaches in the management of infants with congenital diaphragmatic hernia (CDH), mortality rates remain high for those with severe defects.^1^ Recently, research was focused on pulmonary hypertension (PH) and ventricular dysfunction as important contributors to mortality in these infants.^2^ Research of cardiac biomarkers as indicators of disease severity and risk assessment in infants with CDH is gaining popularity. The identification of early cardiac biomarkers is essential, as an optimization of initial drug therapy, the fast implementation of extracorporeal membrane oxygenation (ECMO), or transition to ECMO centers can improve care and outcome of infants with CDH. Several studies focused on the evaluation of brain natriuretic peptide (BNP) and N-terminal pro-BNP (NTproBNP) as cardiac biomarker in infants with CDH.^3–7^ However, studies concerning biomarkers with alternative pathophysiological pathways are needed, because of inconsistent data regarding natriuretic peptides in CDH and possible intra- and inter-individual fluctuations in the first days of life.^4^

The plasma carbohydrate antigen 125 (CA125) demonstrates an increased expression in adult and pediatric patients suffering from heart failure and PH.^8,9^ The high molecular weight glycoprotein CA125 is synthesized by epithelial serous cells. CA125 presents a complex molecular structure with an intracytoplasmic transmembrane domain and an extracellular domain consisting of oligosaccharide chains.^10^ As a tumor marker, CA125 is well established to monitor therapeutic effects in ovarian cancer.^11^ The mechanism of CA125 elevation in heart failure and PH can be explained by different mechanisms. As CA125 is a soluble glycoprotein, it can be released from tissue surfaces or produced by epithelial cells in response to stimuli as mechanical stress and inflammation.^12,13^ Patients with heart failure and signs of congestion are at risk for serosal effusion of the pleura, pericardium, or peritoneum, which potentially stimulates CA125 release from epithelial cells.^14,15^ Furthermore, tumor necrosis factor (TNF)-α, interleukin (IL)-6, and IL-10 were found to correlate significantly with CA125 levels in patients with heart failure.^12^ It is well established that cytokines have a potential role in the pathophysiology of heart failure and cytokines can have negative inotropic effects on cardiac function.^16,17^ The link between inflammatory cytokines and disease severity in infants with CDH was recently evaluated.^18^ Studies in adults revealed that CA125 correlates significantly with mortality in patients with acute heart failure and is associated with the hemodynamic status and echocardiographic parameters.^19–23^ However, research in the pediatric field is scarce.^8^ CA125 seems to be an ideal candidate as biomarker in infants with CDH because it is widely available at economic costs and due to its potential to mirror both cardiac function and patient’s outcome. The aim of this study was to identify CA125 as a useful predictor for PH and ventricular dysfunction in infants with CDH. Furthermore, the study aimed to evaluate CA125 as a predictor for ECMO and mortality in these infants.

## Materials and methods

### Study population and ethic approval

Infants with CDH admitted to the NICU at the University Children’s Hospital of Bonn, Germany in the study period from 06/2018 to 02/2021 were eligible for study participation. Patients were prospectively enrolled in the study after informed written consent was obtained from the parents or legal representative. The study was approved by the local ethics committee of the Medical Center of the University of Bonn (local EC study number 247-16). Infants with an underlying congenital heart defect or primary palliative care were not included in the study.

### CDH management

Prenatal assessment and risk stratification included estimation of the lung size using the observed-to-expected lung-to-head ratio and liver position on ultrasound (measured at 27–29 weeks’ of gestational age (GA)). Right- and left-sided CDH and infants with intrauterine fetal endoluminal tracheal occlusion procedure were included in the analysis. Postnatal CDH management was performed according to the recommendations of the CDH Euro Consortium group. Treatment consisted of the following key strategies: (I) lung-protective ventilation, (II) treatment of PH and right-ventricular, left-ventricular, or biventricular dysfunction (RVD/LVD/BVD), (III) in case of oxygenation failure implementation of veno-venous extracorporeal membrane oxygenation (ECMO), and (IV) delayed surgical repair.^1^

Criteria for ECMO therapy were defined as follows: (a) preductal oxygen saturation <85% or postductal saturation <70%, (b) oxygenation index (OI) ≥ 40 consistently present, (c) PaCO_2_ > 70 mmHg with pH <7.15, (d) peak inspiratory pressure ≥28 cmH_2_O or mean airway pressure ≥17 cmH_2_O, (e) systemic hypotension resistant to fluid and inotropic therapy.^1^

### CA125 measurements

Blood samples for CA125 analysis were collected at the following timepoints after birth: 6, 12, 24, and 48 h. In cases where ECMO was required, additional blood samples were collected daily from day 1 to day 7 during veno-venous ECMO. Blood samples were collected via an indwelling arterial catheter. CA125 serum levels (unit: U/ml) were measured using the electrochemiluminescence immunoassay (Elecsys CA 125 II Assay) on cobas e 801 analyzer (Roche Diagnostics, Basel, Switzerland).

### Echocardiographic assessment

Echocardiography was performed using a Philips CX50 Compact Extreme Ultrasound system with a S12‐4 sector array transducer (Philips Healthcare, Best, The Netherlands). For echocardiographic assessment of PH and RVD/LVD/BVD, standardized measurements at 1–4 h after birth (initial) and 48 h after birth were analyzed. All echocardiographic data were reviewed independently by two neonatal echocardiographers blinded to the clinical course of the respective infants.

PH was graded as following: (I) none, (II) <2/3 systemic pressure (mild), (III) 2/3 to systemic systolic pressure (moderate), and (IV) suprasystemic systolic pressure (severe). Echocardiographic PH evaluation included: (a) ductus arteriosus flow direction, (b) the intraventricular septum position, and (c) the tricuspid valve regurgitation. RVD, LVD, and BVD was assessed separately using a combination of qualitative and quantitative measures based on international guidelines.^24,25^ Ventricular systolic function was graded based on the subsequent quantitative information, if available: fraction shortening, S’ velocity on tissue Doppler imaging, mitral or tricuspid annular plane systolic excursion (TAPSE), or ventricular size, and output.

### Subgroup analysis, outcome parameters, and statistical analysis

Patients were allocated to one of the two subgroups according to the primary endpoint: (group A) need for ECMO or early death within 48 h after birth (despite fulfilling ECMO criteria); (group B) no need for ECMO. Secondary endpoints included in-hospital mortality and length of mechanical ventilation (MV).

Demographic data and baseline characteristics are presented as median with interquartile range or absolute number (*n*) with percentage. Using receiver operating characteristic (ROC) curve analysis, a CA125 cut-off value was evaluated to predict the secondary clinical endpoint (in-hospital mortality). For comparison of continuous variables, Mann–Whitney *U*-test was used. For categorical variables, Fisher’s exact test and chi-square test was applied. Correlations between variables were evaluated by Pearson correlation coefficients and were only applied if any two measurements were taken at the same point in time. A *p* value <0.05 was considered significant. The statistical analysis was performed using the statistical software (IBM SPSS Statistics for Windows, Version 25.0., IBM Corp, Armonk, NY).

## Results

### Study cohort

**Fig. 1:**
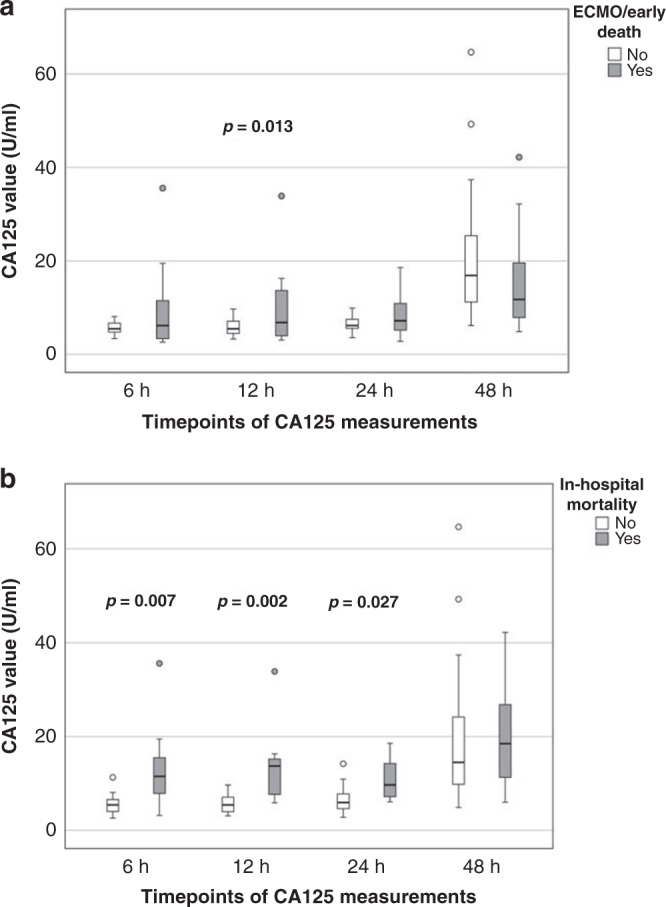
Analysis of CA125 values and outcome parameters. Illustration of CA125 values during first 48 h and subgroup analysis regarding the following parameters: **a** ECMO/early death and **b** in-hospital mortality. *p* Values <0.05 are highlighted in the figure.

Sixty-eight infants (6 infants outborn) with CDH were prospectively enrolled in this study. Demographic data are summarized in Table 1. Overall, ECMO rate and mortality were 38 and 25%, respectively. After group stratification, 28 infants (41%) were allocated to group A and 40 infants (59%) to group B. CA125 values at blood sample timepoints and differences between subgroups are shown in Fig. 1. In group A, CA125 values were significantly higher at 12 h (*p* = 0.013) as compared to infants allocated to group B. When analyzing the secondary endpoint in-hospital mortality separately, CA125 values were significantly higher at day 1 (6, 12, and 24 h: *p* = 0.007, *p* = 0.002, and *p* = 0.027, respectively) in those patients who died.

**Table 1:** Demographic and treatment data.

Variables	CDH cohort *n* = 68	Group A *n* = 28	Group B *n* = 40	*p* level
Gestational age, weeks	36.8	35.8	37.5	**0.019**
Female sex, *n* (%)	33 (49)	14 (50)	19 (48)	0.84
Birth weight, g	2784	2588	2921	0.092
Left-sided CDH, *n* (%)	60 (88)	25 (89)	35 (88)	0.822
o/e LHR, %	39	35	42	**0.034**
Liver herniation, *n* (%)	38 (57)	20 (74)	18 (45)	**0.018**
FETO, *n* (%)	11 (16)	5 (18)	6 (15)	0.670
Diaphragmatic repair, DOL	6.7	7.1	6.4	0.179
Mechanical ventilation, days	17	9	36	**0.000**
In-house treatment, days	52	69	39	**0.022**
In-hospital mortality, *n* (%)	17 (25)	15 (54)	2 (5)	**0.000**
ECMO support, *n* (%)	26 (38)	26 (93)	0	**0.000**
ECMO initiation, h of life		18	8/34	
ECMO duration, days		8.2	3.8/21.8	

Data are demonstrated as absolute number with percentage or as mean values. Data regarding ECMO therapy are demonstrated as median with IQR.

*CDH* congenital diaphragmatic hernia, *DOL* day of life, *ECMO* extracorporeal membrane oxygenation, *FETO* fetoscopic endoluminal tracheal occlusion, *o/e LHR* observed-to-expected lung-to-head ratio.

**Fig. 2:**
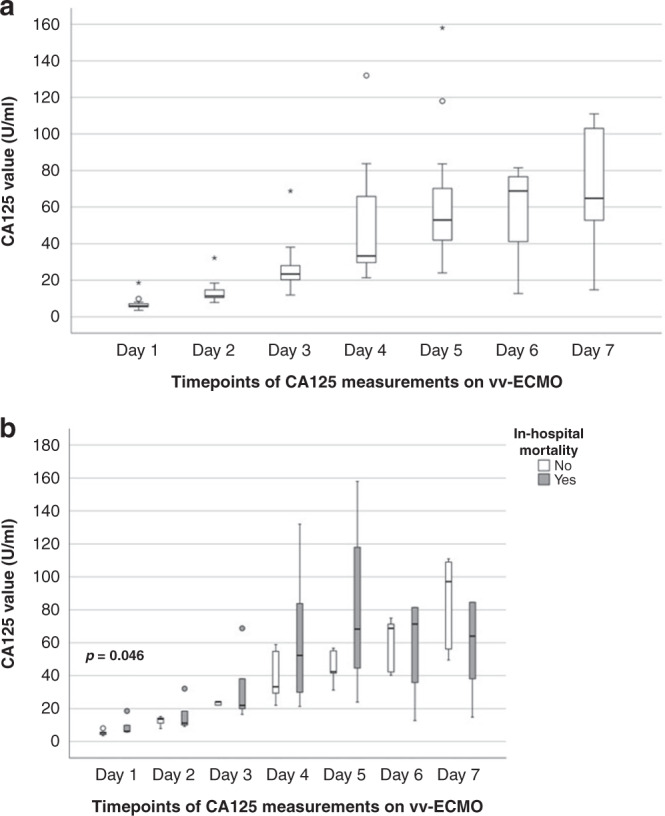
Analysis of CA125 values and ECMO treatment. Illustration of CA125 values during ECMO (**a**) and subgroup analysis of in-hospital mortality (**b**) after weaning from ECMO (prior to discharge). *p* Values <0.05 are highlighted in the figure. Mild statistical outliers are marked with a sphere (°) and extreme outliers with an asterisk (*).

For 24 patients, daily CA125 measurements during ECMO are displayed in Fig. 2. During ECMO, CA125 values increased significantly from day 1 to day 7 in all patients regardless of outcome. ECMO non-survivors presented significantly higher CA125 values on day 1 of ECMO (*p* = 0.046) compared to ECMO survivors. At later timepoints, CA125 values did not differ significantly between subgroups.

Correlation analysis of outcome parameter, baseline characteristics, and cardiac assessment are illustrated in Table 2. CA125 values measured at day 1 (6, 12, 24 h) correlated significantly with length of MV. When comparing CA125 values with baseline characteristics, a significant negative correlation was detected for GA (at 12 and 24 h) and a significant positive correlation for infants with liver herniation (at 12 h).

**Table 2:** Correlation analysis of CA125 serum values with outcome parameters, baseline characteristics, and cardiac assessment.

CA125	6 h correlation (*p* level)	12 h correlation (*p* level)	24 h correlation (*p* level)	48 h correlation (*p* level)
*Outcome parameter*				
Length of MV	**0.339 (0.05)**	**0.480 (0.01)**	**0.306 (0.04)**	0.103 (0.48)
*Baseline characteristics*				
Gestational age	−0.188 (0.228)	−**0.317 (0.016)**	−**0.311 (0.017)**	−0.185 (0.157)
Birth weight	−0.089 (0.572)	−0.258 (0.053)	−0.091 (0.493)	−0.039 (0.768)
o/ e LHR	0.121 (0.456)	0.014 (0.922)	−0.075 (0.602)	0.217 (0.123)
Liver herniation	0.260 (0.097)	**0.269 (0.045)**	0.251 (0.058)	0.016 (0.902)
FETO	−0.089 (0.570)	0.046 (0.732)	−0.026 (0.843)	0.083 (0.529)
*Cardiac assessment*				
PH				
Admission	**0.312 (0.04)**	**0.370 (0.05)**	0.232 (0.08)	0.094 (0.48)
48 h	0.248 (0.11)	0.230 (0.09)	−0.22 (0.86)	−0.17 (0.21)
RVD				
Admission	−0.032 (0.82)	−0.031 (0.82)	**0.264 (0.04)**	0.201 (0.13)
48 h	−0.157 (0.32)	−0.128 (0.35)	−0.117 (0.39)	−0.234 (0.07)
LVD				
Admission	0.000 (1.00)	0.003 (0.98)	0.079 (0.55)	**0.333 (0.01)**
48 h	−0.110 (0.49)	−0.089 (0.52)	−0.155 (0.25)	−0.057 (0.67)
BVD				
Admission	0.278 (0.07)	**0.269 (0.04)**	0.111 (0.40)	−0.115 (0.39)
48 h	**0.503 (0.00)**	**0.580 (0.00)**	0.082 (0.54)	−0.107 (0.42)

Pearson correlation was performed for calculation of the correlation coefficient. *p* Levels <0.05 are highlighted in bold.

*BVD* biventricular dysfunction, *ECMO* extracorporeal membrane oxygenation, *FETO* fetoscopic endoluminal tracheal occlusion, *LVD* left ventricular dysfunction, *MV* mechanical ventilation, *o/e LHR* observed-to-expected lung-to-head ratio, *PH* pulmonary hypertension, *RVD* right ventricular dysfunction.

**Fig. 3:**
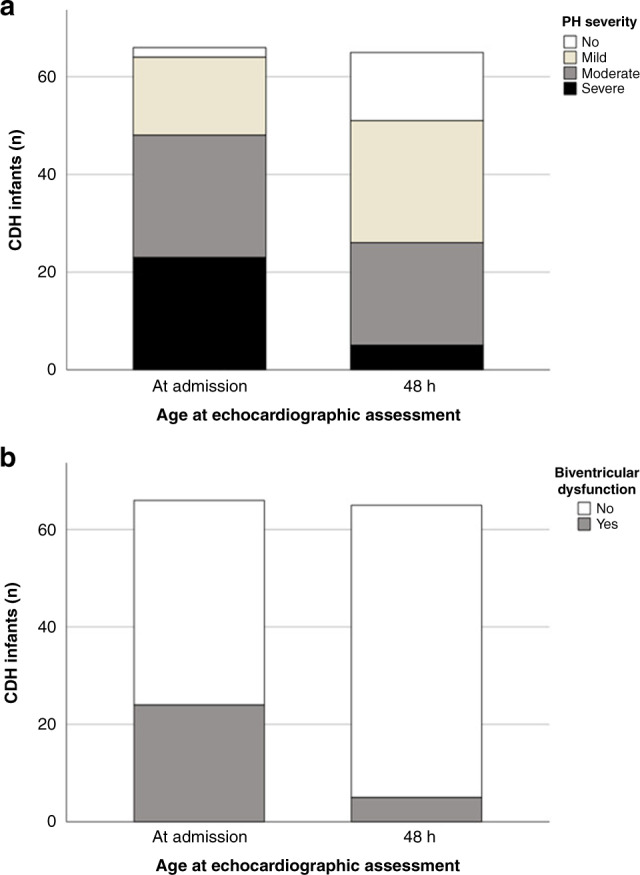
Echocardiographic assessment. Illustration of echocardiographic evaluation of PH severity (**a**) and biventricular dysfunction (**b**).

Prevalence and course of PH severity and ventricular dysfunction on admission and at 48 h are displayed in Fig. 3. PH was prevalent in 94% of infants with CDH on admission and prevalence decreased to 76% at 48 h. Most infants presented with a combination of PH and RVD (70% on admission, 65% at 48 h). No infant had signs of isolated RVD at both timepoints. One infant presented an isolated LVD at 48 h. PH severity on admission correlated significantly with mean CA125 values at 6 and 12 h (see Table 2). When looking only for infants with severe PH on admission, these infants presented significantly higher mean CA125 values at 12 h (10.6 vs. 5.8 U/ml, *p* = 0.004) compared to infants with none–severe PH (mild or moderate). This trend was not seen in PH assessment at 48 h. When analyzing infants presenting an isolated PH (without RVD) on admission (23 infants), PH did not correlate significantly with mean CA125 values at 6 and 12 h. RVD on admission showed significant correlation with CA125 values at 24 h, without significant findings at the other timepoints of CA125 measurement. In contrast, the presence of LVD on admission was found to correlate significantly with mean CA125 values at 48 h. Mean CA125 values measured at 6 and 12 h were significantly correlated with BVD.

**Fig. 4:**
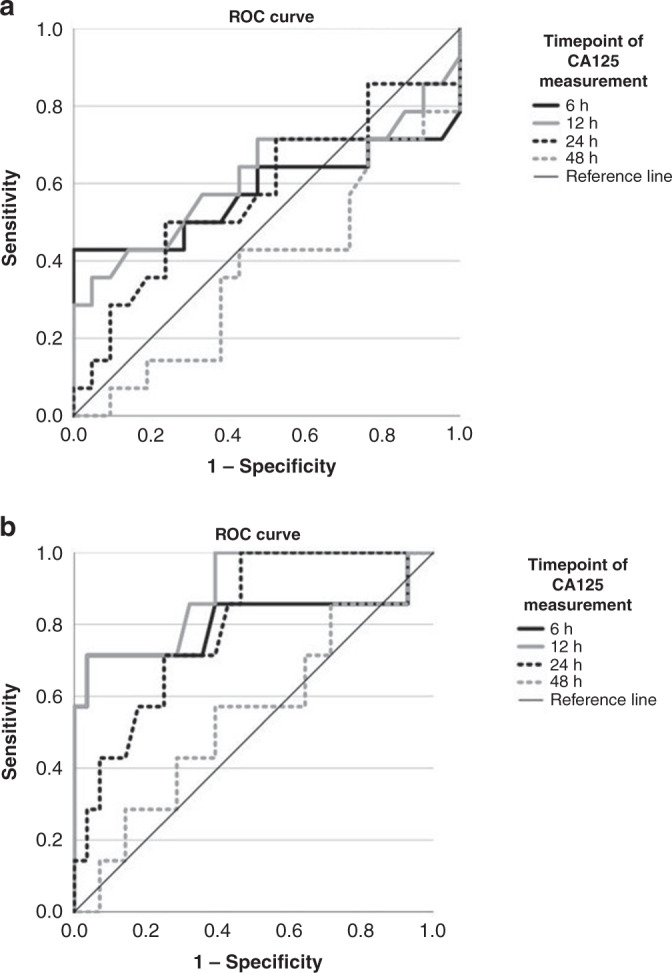
Illustration of ROC analysis for outcome parameters. ROC curve analysis of CA125 values regarding the primary endpoint ECMO/early death (**a**) and the secondary endpoint in-hospital mortality (**b**).

In all, 100% of the infants presenting a CA125 value ≥10 U/ml at 6 h of life had subsequent need for ECMO (*p* = 0.001). Additionally, 86% of the infants with a CA125 value ≥10 U/ml at 6 h did not survive to discharge (*p* = 0.001). The ROC curves of CA125 measurement at 6, 12, 24, and 48 h to predict ECMO/early death and in-hospital mortality are displayed in Fig. 4. The area under the curve (AUC), sensitivity, specificity, and positive as well as negative predictive values (PPV and NPV, respectively) for a CA125 cut-off value ≥10 U/ml are illustrated in Table 3. The highest AUC values were calculated for in-hospital mortality at 6, 12, and 24 h. The best combination of NPV and PPV for ECMO/early death was shown at 6 and 12 h, with a PPV of 100% for both timepoints and a NPV of 70, and 68%, respectively. Accordingly, for in-hospital mortality the highest PPV was shown at 6 and 12 h (83 and 100%, respectively), with a NPV of 87 and 88%, respectively.

**Table 3:** ROC analysis for outcome parameters.

CA125 cut-off ≥10 U/ml	6 h	12 h	24 h	48 h
*Outcome parameter*				
ECMO/early death, AUC (*p* level)	0.578 (0.503)	0.617 (0.210)	0.578 (0.455)	0.379 (0.223)
Sensitivity, %	36	29	29	71
Specificity, %	100	100	91	24
PPV, %	100	100	68	38
NPV, %	70	68	66	55
In-hospital mortality, AUC (*p* level)	**0.809 (0.014)**	**0.895 (0.000)**	**0.801 (0.000)**	0.546 (0.714)
Sensitivity, %	57	57	43	86
Specificity, %	96	100	90	29
PPV, %	83	100	59	29
NPV, %	87	88	83	86

ROC analysis was performed for calculation of the area under the curve (AUC). *p* Levels <0.05 are highlighted in bold.

*ECMO* extracorporeal membrane oxygenation, *NPV* negative predictive value, *PPV* positive predictive value.

## Discussion

This is the first study evaluating serial measurements of the biomarker CA125 in the first 48 h of life and during ECMO in infants with CDH. A major aim of this study was to evaluate the glycoprotein CA125 as a biomarker of the cardiac status in infants with CDH. According to our data, CA125 values correlated significantly with both PH severity and ventricular dysfunction. In the past decade, the potential of CA125 as a prognostic biomarker in adults with acute and chronic heart failure was evaluated.^9,20,26–29^ CA125 is associated with PH severity and echocardiographic markers of right- and left-ventricular function in adult populations.^21–23,29,30^ Studies analyzing CA125 as a biomarker for cardiac function in newborns or children are scarce. Pektas and colleagues evaluated CA125 in a cohort of 25 children (median age 7.5 years) with secondary PH and demonstrated that a CA125 cut-off >35 U/ml was highly sensitive and specific for the presence of PH.^8^ Children without heart disease and PH findings (control group of 40 healthy children) presented with significantly lower CA125 levels with a mean value of 13.8 U/ml. CA125 values were found to correlate significantly with echocardiographic markers of RVD (right-ventricular fractional area change and TAPSE values). Similarly, we could demonstrate a significant correlation between CA125 values and echocardiographic markers of PH as well as ventricular dysfunction. But this association was not evident in infants with CDH presenting PH without signs of ventricular dysfunction, assuming that ventricular dysfunction seems to be the major determinant for CA125 release. Infants with PH without ventricular dysfunction presented overall a mild course of PH and CA125 seems to be ineffective as a biomarker in this subgroup.

The second aim of this study was to prove CA125 as a predictor of patient’s outcome. Our data revealed that CA125 seems to be useful as an early predictor for the need of future ECMO in CDH infants and to identify infants at risk for in-hospital mortality. In most infants, ECMO was initiated after 12 h of life (median 18 h). Therefore, the early measurement of CA125 could be a relevant tool for guidance of vasoactive therapy, the decision to evaluate ECMO indication, or to transfer infants to an ECMO center when ECMO is not available. Further studies aiming to analyze CA125 for risk stratification of ECMO are lacking. In our cohort, infants with a need for ECMO had a lower GA than infants not requiring ECMO. As CA125 values seems to correlate negatively with GA, data regarding CA125 measurements during ECMO need to be interpreted carefully.

Longitudinal CA125 measurement during ECMO in our study were intended to early identify infants with CDH who can be successfully weaned from ECMO and infants who will fail to wean from ECMO. In our cohort, CA125 values measured during ECMO tended to increase over time from day 1 to day 7, which is a contrary finding to the observed improvement of PH and ventricular dysfunction during ECMO. Only values measured on day 1 during ECMO differed significantly between ECMO survivors and non-survivors, with significantly higher CA125 values in infants who failed to wean from ECMO. Therefore, only early CA125 measurements at the beginning of ECMO rather than longitudinal CA125 measurements during ECMO seems reasonable. Infants during ECMO are likely to present a systemic inflammatory response syndrome, with capillary leak and fluid overload, causing pleural effusions and ascites. Additionally, blood flow via the return cannula during ECMO lead to increased right atrial and central venous pressures, inducing changes in the blood distribution of the central venous system and splanchnic veins. These changes potentially influence the CA125 release from epithelial cells and lead to an increase of CA125 values seen during ECMO.

Standardized reference values for CA125 in infants are available from one study, with 105 cord blood samples from preterm and 28 samples from term infants.^31^ Preterm infants aged 24–32 weeks’ GA presented mean CA125 values of 68 U/ml, infants aged 33–37 weeks’ GA presented mean CA125 values of 34 U/ml, and term infants (>37 weeks’ of GA) presented mean values of 10 U/ml. These findings are in line with our data, presenting a negative correlation of the GA and CA125. Two explanations are possible. First, CA125 is highly expressed by fetal/placental tissues (e.g., celomic epithelium as pleura, peritoneum, pericardium; derivatives of the Mueller epithelium; and the amnion) and levels are high during rapid fetal growth in early phases of GA, with a decreasing expression to later phases of gestation.^32^ Second, immaturity of the fetal hepatic clearance of glycoproteins (as CA125) is leading to higher CA125 concentrations in earlier stages of gestation.^31^ CA125 between the first year of life seems to remain at low levels in comparison to cord blood samples of term infants. Data of this Finnish cohort are not comparable one to one with our data, because all blood samples were taken in infants with CDH and cord blood samples were not analyzed. But the data emphasize that a CA125 value ≥10 U/ml in newborn infants with CDH is a reasonable cut-off when comparing both data sets.

CA125 levels at 12 h were found to correlate significantly with liver herniation in our cohort. As liver herniation is a marker of disease severity and is associated with higher incidences of ventricular dysfunction and PH, these infants potentially express higher CA125 values due to right-ventricular fluid overload, causing central venous congestion and ascites. In case of LVD with high postcapillary pressures, infants tend to produce more pleural effusions, leading to CA125 elevation.

When comparing CA125 with established biomarker such as natriuretic peptides, CA125 seems to have potential advantages in prognosing long-term mortality and RVD in patients with acute heart failure.^21^ CA125 seems to outperform natriuretic peptides in patients with combined left and right heart failure. Additionally, CA125 values are not altered in patients with renal impairment and therefore provide better information in cases of acute or chronic renal failure, as recently shown in large adult populations.^33,34^ Other data suggest that the risk stratification in acute heart failure can be optimized when combining CA125 with natriuretic peptides.^19^ A combination of both parameters might be a reasonable approach in a neonatal cohort for future studies.

In summary, CA125 seems to be an ideal biomarker to monitor the clinical course of infants with CDH, as it correlates significantly with PH severity and ventricular dysfunction. Additionally, a CA125 cut-off value of ≥10 U/ml at 6 and 12 h is highly predictive for ECMO and in-hospital mortality. Future prospective trials are required to further analyze CA125 as a biomarker, because it could potentially help to optimize initial treatment and could be a helpful clinical tool for low-volume as well as high-volume CDH treatment centers.

## Limitations

Due to study design, this study has some limitations. A comparative cohort is missing, because multiple laboratory assessments and at least two echocardiographic assessments in 48 h were performed, which raises problems of ethical and parental approval in a healthy control group. Nevertheless, a comparison group would facilitate the interpretation of results and statistical effects. The echocardiographic assessment of PH and ventricular dysfunction was based to some extent on a qualitative grading, with the risk of observer-related bias. We tried to minimize this bias by reviewing the echocardiographic data independently by two neonatal echocardiographers, blinded to the clinical course of the respective subject.

## Conclusion

This is the first prospective study evaluating CA125 as a cardiac biomarker in a cohort of infants with CDH. Our data demonstrate a correlation of CA125 with PH and ventricular dysfunction and an increased expression in infants with a more complicated hospital course or poor outcome (need for ECMO, in-hospital mortality, duration of MV).

## Data Availability

All data generated or analyzed during this study are included in this published article [and its Supplementary Information files].
